# Threading-Induced Dynamical Transition in Tadpole-Shaped Polymers

**DOI:** 10.1021/acsmacrolett.0c00197

**Published:** 2020-05-05

**Authors:** Angelo Rosa, Jan Smrek, Matthew S. Turner, Davide Michieletto

**Affiliations:** † SISSA (Scuola Internazionale Superiore di Studi Avanzati), Via Bonomea 265, 34136 Trieste, Italy; ‡ Faculty of Physics, University of Vienna, Boltzmanngasse 5, A-1090 Vienna, Austria; § Department of Physics and Centre for Complexity Science, University of Warwick, Coventry CV4 7AL, United Kingdom; ∥ Department of Chemical Engineering, Kyoto University, Kyoto 606-8501, Japan; ⊥ School of Physics and Astronomy, University of Edinburgh, Peter Guthrie Tait Road, Edinburgh EH9 3FD, United Kingdom; # MRC Human Genetics Unit, Institute of Genetics and Molecular Medicine, University of Edinburgh, Edinburgh EH4 2XU, United Kingdom; ∇ Department of Mathematical Sciences, University of Bath, North Rd, Bath BA2 7AY, United Kingdom

## Abstract

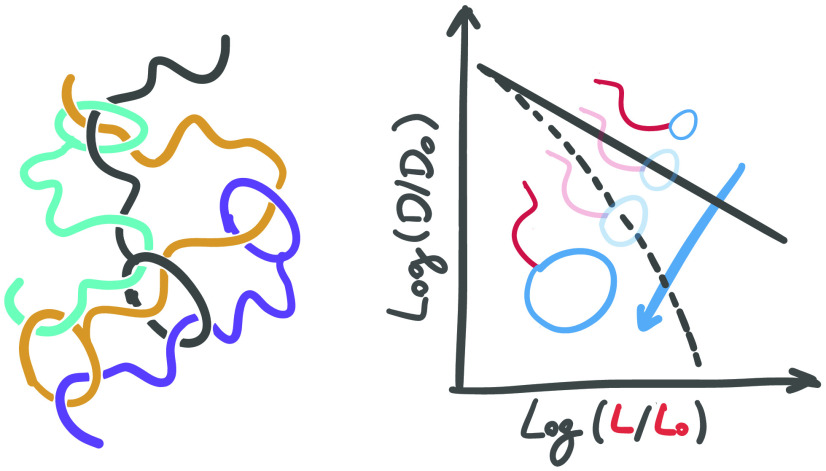

The relationship between polymer topology and bulk rheology remains a key question in soft matter physics. Architecture-specific constraints (or threadings) are thought to control the dynamics of ring polymers in ring–linear blends, which thus affects the viscosity to range between that of the pure rings and a value larger, but still comparable to, that of the pure linear melt. Here we consider qualitatively different systems of linear and ring polymers, fused together in “chimeric” architectures. The simplest example of this family is a “tadpole”-shaped polymer, a single ring fused to the end of a single linear chain. We show that polymers with this architecture display a threading-induced dynamical transition that substantially slows chain relaxation. Our findings shed light on how threadings control dynamics and may inform design principles for chimeric polymers with topologically tunable bulk rheological properties.

The tube and reptation theories underpin our understanding of complex fluids.^1,2^ However, the seemingly innocuous joining of the polymers’ ends to form rings poses a problem that has been puzzling the polymer physics community for over three decades.^3−22^ How do topology-specific constraints affect the static and dynamic properties of a dense solution of such polymers?

Entangled solutions of pure unlinked ring polymers can now be synthesized.^11,23^ However, the presence of even a small fraction of linear contaminants dramatically slows their dynamics through ring–linear interpenetration.^11,24−27^ This slowing down shares some similarities with the one computationally discovered in systems of pure rings,^28−31^ where inter-ring threadings drive a “topological glass” state due to a hierarchical network of threadings: ring-specific topological constraints.^32−36^ In ring–linear blends, the linear chains cannot set up a hierarchical network of constraints and the rings are thus bound to relax on time scales comparable to the reptative disengagement of the linear chains^4,37−39^ which perform most of the threadings: this limits severely any opportunities for further tuning of bulk rheology by using pure mixtures of ring and linear chains.

To overcome this limitation, and inspired by quickly progressing technical advances in topological polymer synthesis,^40−42^ here we investigate the behavior of polymer architectures that simultaneously display linear and unknotted and unlinked circular topologies. We dub these architectures “chimeric,” the name given to any mythical animal formed from parts of various other animals (Figure 1A). The simplest example of a chimeric architecture is that of a tadpole-shaped polymer, “tadpole” for brevity (see Figure 1B,C), which has recently been realized experimentally^43,44^ and has attracted considerable attention in the field of protein folding.^45,46^

**Figure 1:**
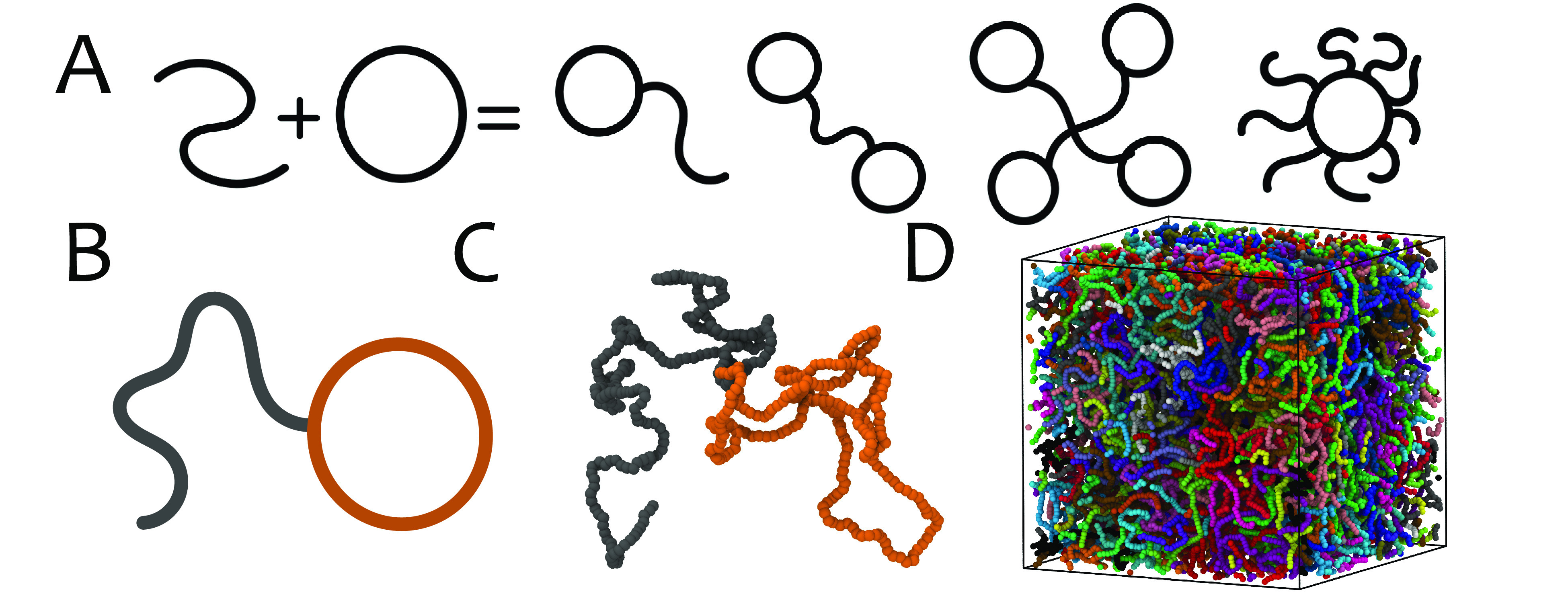
(A) Chimeric polymers from ring and linear chains fused together. (B) Tadpole-shaped polymers are the simplest such chimeric structure, shown as a schematic with orange “head” and gray “tail.” (C) Typical simulated conformation of a tadpole and (D) an equilibrated system of 80 tadpoles. Here the circular and linear sections both have 250 monomers, written (*C*, *L*) = (250, 250).

While a broader class of polymers (dubbed “topological”) has been studied in dilute conditions,^47,48^ in this Letter, we focus on entangled, semidilute concentrations and report the first molecular dynamics simulation (Figure 1D) of tadpole-shaped polymers in this regime. Our main finding is that we observe a dynamical transition in which systems of tadpoles with long enough tails and heads display a markedly slower dynamics than a corresponding system of linear chains with equal mass. This extremely slow dynamics is expected to arise only at asymptotically large lengths in systems of pure rings,^33,36^ while it cannot be achieved in standard blends of ring and linear chains^11,26,37,38^ where only a ∼2-fold increase in viscosity has been reported;^4,39^ while in blends there is no strategy to slow down the linear fraction beyond their natural reptative dynamics, in tadpoles this is achieved by a system-spanning (percolating) set of topological constraints that propagate from the ring to the linear part due to the permanent junction.

To study tadpole microrheology, we model tadpole-shaped polymers as bead–spring chains made of a “tail” (linear) and a “head” (circular) components. The monomers are connected by finitely extensible (FENE), bonds and we impose a persistence length *l*_p_ = 5σ, with σ being the size of a monomer, via a Kratky–Porod potential (see the Supporting Information (SI)). The junction between head and tail is fully flexible, and we consider athermal solvents in which the beads interact via a purely repulsive Lennard–Jones (WCA) potential.^49^ The systems are made of *M* chains with *N* beads each at the overall monomer density ρ = *NM*/*V* = 0.1σ^–3^ (about 10 times the overlap concentration). With these choices, the corresponding entanglement length for a system of linear chains is *N*_e_ = 40 beads;^15,50^ our longest tadpoles have tails 10*N*_e_ long, thus putting them well into the entangled regime. The simulations are performed in implicit solvent at fixed volume and temperature by weakly coupling the dynamics of the monomers with a heat bath via LAMMPS.^51^ The Langevin equations are evolved using a velocity-Verlet algorithm with integration step Δ*t* = 0.012τ_LJ_, where τ_LJ_ = σ(*m*/ϵ)^1/2^ is the Lennard–Jones time (see SI).

To characterize the dynamics of the tadpoles, we measure the averaged mean-square displacement (MSD) of their center of mass (CM) as *g*_3_(*t*) = ⟨(*r⃗*_*i*_(*t*_0_ + *t*) – *r⃗*_*i*_(*t*_0_))^2^⟩, where *r⃗*_*i*_(*t*) is the position of the CM of the *i*th tadpole at time *t* and ⟨...⟩ indicates time and ensemble average (see Figure 2A). The trajectories display a subdiffusive regime at short-intermediate times which appears to scale as *g*_3_(*t*) ∼ *t*^0.4^ for our largest tadpoles (we compute the dynamical exponent α(*t*) = *d* log *g*_3_/*d* log *t* in the SI). We note that this scaling exponent is distinct from, and smaller than, that of pure entangled linear chains (*t*^0.5^) and also pure rings (*t*^0.75^),^13^ suggesting that tadpole dynamics appears to follow new physical mechanisms that are distinct from those of polymers with simpler topologies.

**Figure 2:**
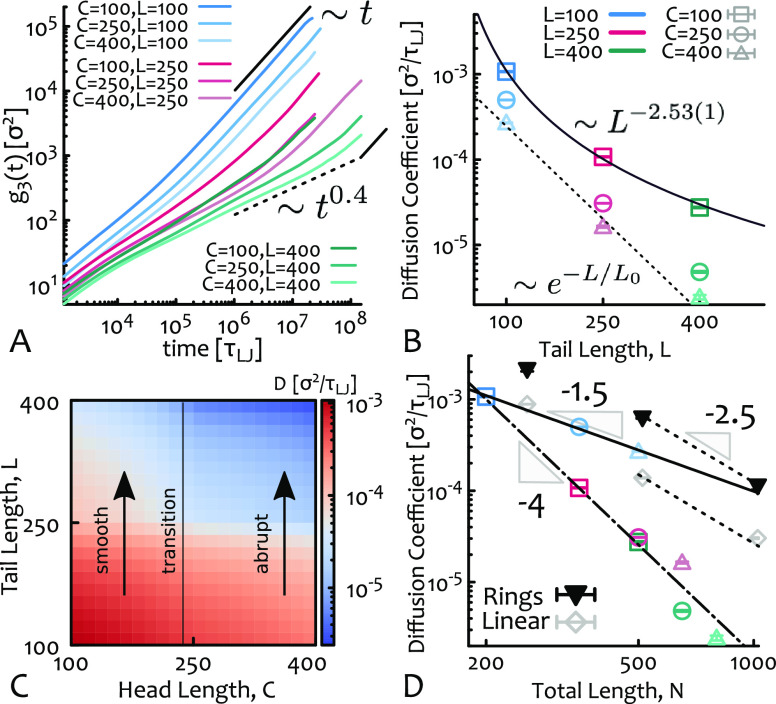
(A) Mean-square displacement of the center of mass, *g*_3_(*t*), of the tadpoles. (B) Log–linear plot of long-time diffusion coefficient *D* against tail length *L*. The data set with *C* = 100 is well fitted by a power law ∼*L*^–*a*^ with *a* = 2.53(1), while tadpoles with larger heads display a qualitatively different slowing down with *a* = *a*(*L*) increasing with tail size and compatible with an exponential (shown as a dashed line as a guide for the eye). (C) Interpolated heat map of *D* in the 2D parameter space (*C*, *L*). (D) Plot of *D* against total contour length and compared with the dynamics of pure linear and ring polymers. The solid, dashed, and dashed-dotted lines are guides for the eye. The dashed line indicates the known scaling for asymptotic ring and linear chains.^13^ Note that *D*(*L* = 400, *C* = 400) is an upper bound value as the system has not reached free diffusion within our longest simulation runtime.

To quantify how the dynamics varies with tadpole design, we compute the large-time diffusion coefficient of the center of mass as *D* = lim_*t*→∞_*g*_3_(*t*)/6*t* (i.e., we constrain the dynamical exponent α = 1 and choose a time range for which this is accurate; see the SI) and plot it as a function of tail length in Figure 2B. From this, one should notice that the different designs display qualitatively different behaviors: for a small head *C* = 100, the slowing down with tail length (*L*) is well fitted by a power law *D* ∼ *L*^–2.53(1)^ similar to that of pure reptating linear chains.^13,49^ This suggests that the interactions between tails dominates the dynamics in this case; on the other hand, the two sets of simulations with *C* = 250 and *C* = 400 display a qualitatively different scaling behavior whereby *D* ∼ *L*^–*a*^ with *a* > 3 and increases with *L*, yielding a dynamics slower than reptation. Interestingly, comparing the square sum of residuals reveals that these two data sets are better fitted by an exponential, rather than a power law, decay. This change, or transition, in behavior can also be qualitatively visualized in a heat map of *D* as a function of tadpole design (*C*, *L*): *D* decays smoothly for *C* < 250 and more abruptly for *C* > 250 (Figure 2C).

Importantly, as shown in Figure 2D, while the dynamics displayed by the system of tadpoles with *C* = 100 interpolates in between the pure-ring and pure-linear dynamics, the two sets with *C* ≥ 250 are markedly slower and they follow a qualitatively different trend also as a function of total length *N* = *C* + *L*. Thus, our findings strongly suggest that, via targeted design of tadpole structure, and in principle other chimeric architectures, it is possible to achieve a fine control over the bulk rheology and over a range that is orders of magnitude broader than the one that can be achieved using simpler architectures within the same window of polymer length. It should also be highlighted that while adding linear contaminants to solutions of rings only generates systems that interpolate between the pure-ring and pure-linear behaviors,^11,26^ with chimeric polymers, due their fused architecture, we can produce emergent collective behaviors which have no counterpart in ring–linear blends. We now show that these observed collective phenomena are due to intertadpole “threadings,” i.e., piercing of a tadpole’s tail through the head of another.

Motivated by previous work,^28,43,52^ we hypothesize that threadings may give rise to an emergent slowing down in our entangled tadpoles. To identify threadings, we use the concept of minimal surfaces:^21,31,53^ we first fix a boundary using the position of the beads forming the heads and generate an initial triangulated surface; we then evolve this surface via the Surface Evolver under the action of surface tension until the area is minimized.^54^ Once a minimal surface is defined for each tadpole head, we look for intersections between all possible pairs of tail and head surface (see Figure 3A). [We choose to exclude self-intersections as they may be ill-defined in some cases]. This strategy allows us to define a time-dependent threading matrix as follows: *T*_*ij*_(*t*) = 1 if tadpole *j* is threading tadpole *i* (*i* ≠ *j*) and 0 otherwise.

**Figure 3:**
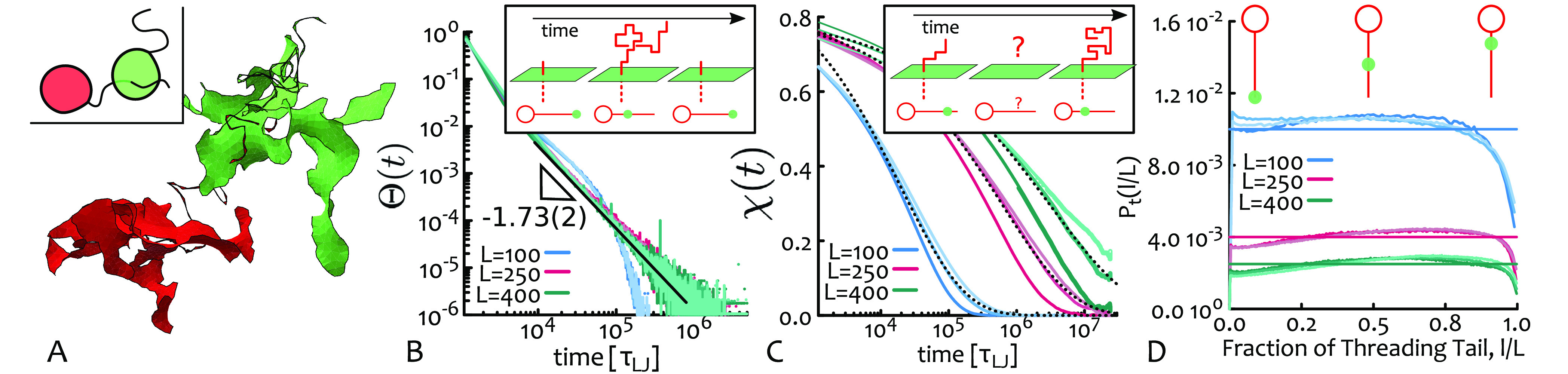
(A) Snapshot of two threading tadpoles with their minimal surfaces highlighted in red and green. (Inset) Sketch of the snapshot. (B) Distribution of return times Θ(*t*) as defined in eq 1 and representative fit ∼ *t*^–β^ with β = 1.74 ± 0.02 for *C* = 250, *L* = 400. (Inset) Mapping to an anomalous Brownian walk in 1D along the tail. (C) Two time-point correlator χ(*t*). Dashed lines are representative stretched exponential fits yielding exponents γ = 0.359(5) for *C* = 250, *L* = 400; γ = 0.416(5) for *C* = 250, *L* = 250; and γ = 0.459(4) for *C* = 250, *L* = 100. (Inset) Graphical sketch of the two-point correlation, stressing that χ(*t*) is insensitive to threading history. (D) Threading lengths are uniformly distributed. The horizontal lines mark inverse tail length, i.e., 1/*L*, for the three sets. The distributions *P*_*t*_ are normalized so that Σ_*l* = 1_^*L*^*P*_*t*_(*l*) = 1.

Threadings are stochastic events that last for a certain time, and we quantify the distribution of these threading lifetimes via the following quantity
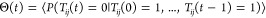
1where *P*(*X*|*Y*) is the probability of observing *X* conditioned on *Y* being observed and ⟨...⟩ indicates the ensemble and time average. In practice, eq 1 counts the threadings with lifetime exactly *t* and the resulting curves are reported in Figure 3B. To discuss these curves, we should note that the eq 1 calculation can be mapped to that of a first return time (or first passage time) of a Brownian Walk in 1D. In this framework, the walker represents the intersection of the tail through the head-spanning minimal surface; the walker moves along the tail as the threading diffuses in and out the minimal surface (see inset of Figure 3B). The distribution of return times of a Brownian walk is expected to be a power law and to scale as ∼*t*^α/2–2^, where α is the anomalous exponent of the walk.^55,56^ In our case, the tails are expected to follow a Rouse dynamics, confirmed by direct tracking of the piercing segment, which yields α = [0.4, 0.6] (see SI), and we thus predict the distribution of return times to scale with an exponent α/2–2 = [1.7, 1.8] in very good agreement with our best fits of Θ(*t*) for *L* ≥ 250 (see Figure 3B). [The curves with *L* = 100 display a scaling exponent closer to −1.5 as their Rouse regime is shorter than our sampling time.]

Importantly, we note that the slowest return time displayed by Θ(*t*) is still ∼10-fold faster than the longest relaxation of the tadpoles (10^6^τ_LJ_ versus 10^7^τ_LJ_, compare the curves Θ with the crossover time to diffusion of *g*_3_ in Figure 3B and Figure 2A, respectively). This suggests that it is collective multithreading events that control the long-time dynamics of tadpoles.

In light of this, we study the two time-points correlator χ(*t*) = ⟨*T*_*ij*_(*t*) *T*_*ij*_(*t + t*_0_))⟩ – *p*_*T*_, where *p*_*T*_ = ⟨ϕ⟩/(*M* – 1) is the background probability that any two tadpoles are threading at any given time and ⟨...⟩ is the average over times *t*_0_ and pairs of tadpoles (*i*, *j*). We note that the longest relaxation time of χ(*t*), i.e., the time at which χ ≃ 0, broadly agrees with the crossover time to free diffusion of the tadpoles (compare Figure 4C with Figure 2A). This quantity is akin to a stress relaxation in polymeric systems and informs us on the relaxation dynamics of intertadpole threadings. By assuming that threadings are monodisperse in length, we would expect χ(*t*) ∼ e^–*t*/*T*(*l*)^, where *T*(*l*) is the typical relaxation time of a threading of length *l*. Instead, we find that χ(*t*) decreases as a stretched exponential χ(*t*) ∼ exp(−*At*^γ^) as expected for a polydisperse solution of entangled linear polymers.^57^ In the case of polymer lengths that follow a Poisson distribution, the exponent γ can be computed via a saddle point approximation to be γ = 1/(1 + β) (where β = 2 and 3 for Rouse and reptation, respectively).^57,58^ In our case, we find that the distribution of threading lengths, i.e., the portion of tail from the piercing point to the end of the tail, is instead uniform, i.e., *P_t_*(*l*) ≃ 1/*L* (see Figure 3D). Thus, to compute their relaxation, we must calculate χ(*t*) = (1/*L*) ∫_0_^*L*^ e^–*t*/*T*(*l*)^ d*l*, where *T*(*l*) = τ_0_*l*^δ^ now depends on the threading length *l* through a generic exponent δ. This function can be computed numerically as a function of τ_0_ and δ for different choices of *C* and *L*. As expected, we find that τ_0_ is overall independent of either *C* or *L* (see SI); on the other hand, we find that δ, which is also expected to be insensitive of *L* within the classic reptation dynamics, increases as a power law of *L* for small heads and exponentially in *L* for large heads (Figure 4A). This implies that *T*(*l*) diverges even more strongly than an exponential in the asymptotic limit of large tadpoles. We should note that the distinct behavior of *T*(*l*) for small and large heads mirrors the qualitatively distinct regimes observed in the decay of *D* (Figure 2B). This strongly suggests that threadings play a key role in the dynamics.

**Figure 4:**
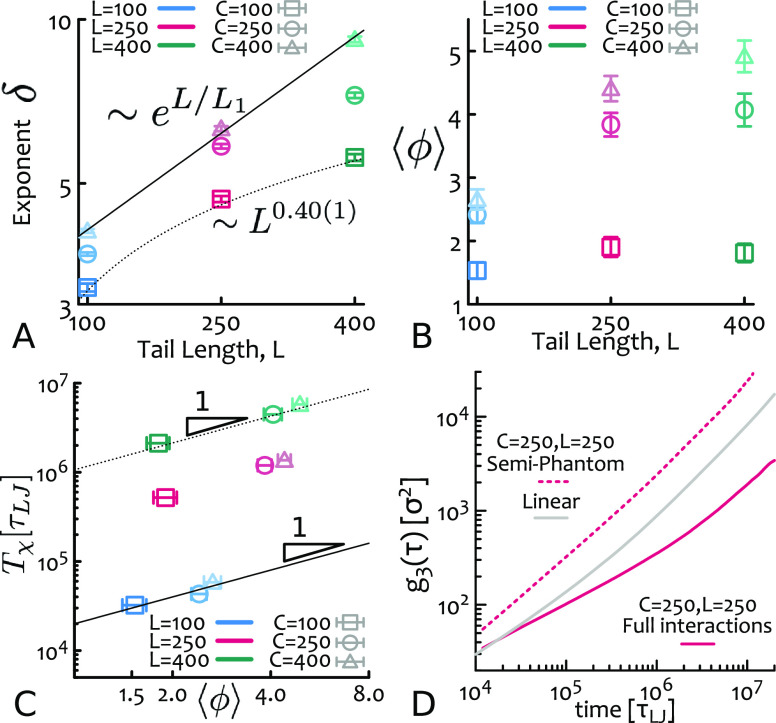
(A) Threading relaxation exponent δ increases with tail length as a power law δ ∼ *L*^0.40(1)^ for small heads and exponentially δ ∼ e^*L*/*L*_1_^ with *L*_1_ for large heads. (B) Average number of threading tails per tadpole ⟨ϕ⟩ as a function of tail length. (C) Threading correlation time *T*_*χ*_ scales linearly with ⟨ϕ⟩ (with a prefactor proprtional to *L*^3^), suggesting that a serial release of ⟨ϕ⟩ threadings is needed before all constraints are released. (D) Comparison of *g*_3_(*t*) in presence and absence of threading constraints (see the text).

The results shown up to now suggest that tadpoles with large heads have a qualitatively different dynamics with respect to the ones with smaller head; in particular, the tadpoles with larger heads display a much stronger slowing down and slower threading relaxation. To explain this finding, we note that the head-spanning minimal surfaces scale linearly^31^ with head length *C* (see also the SI) and, hence, tadpoles with larger heads are expected to accommodate more threadings. In particular, we expect that the number of threadings per head should scale linearly with *C* (and hence with *N*) in the asymptotic limit. From the time-dependent threading matrix *T*_*ij*_(*t*), we can extract the mean number of (passive) threadings per tadpole as ⟨ϕ⟩ ≡ ⟨Σ_*j*≠*i*_*T*_*ij*_(*t*)⟩, where the average is performed over time and tadpoles. This quantity is reported in Figure 4B, and indeed it shows that for small heads the number of threadings is saturated at modest tail lengths; on the other hand, larger heads can accommodate up to five threadings, on average, and often each threading is made by more than one piercing (see SI). Importantly, they appear to saturate at much larger values of tail length and arguably will scale extensively with *L* in the limit of large heads *C*. A natural consequence of the fact that ⟨ϕ⟩ > 1 is that these systems are percolating; i.e., the largest number of tadpoles connected by threadings is comparable with the system size. In particular, we find that the critical threading length required to set up a percolating cluster of tadpoles is *l*_c_/*L* = 1/⟨ϕ⟩ (see SI).

To correlate the mean number of threadings with a dynamical quantity, we extract a characteristic time from χ as *T*_*χ*_ = ∫_0_^∞^χ(*t*) d*t* and find that *T*_χ_ ∼ ⟨ϕ⟩ (Figure 4C), suggesting that the full relaxation of threading constraints depends on the number of threadings. This can be explained by noting that the full relaxation appears to need ⟨ϕ⟩ serial release events before (all) the threading constraints are released. We also note that the diffusion coefficient strongly depends on the mean threading number (see SI). An exact quantification of the variation of tadpole mobility with number of threadings alone is difficult since *D* is also a function of total contour length.

To unambiguously detect the role played by threadings in the dynamics of tadpoles, we thus propose a new strategy: we investigate a symmetric (i.e., *C* = 250, *L* = 250) system of tadpoles with phantom (no steric) interactions between heads and tails while maintaining standard self-avoidance between pairs of monomers belonging to two heads or two tails. This entails that threadings of heads by tails are no longer topological constraints for the dynamics of the tadpoles. To fairly compare with our other results, we compress this system 2-fold (in volume) to maintain the effective (self-avoiding) monomer density at ρ = 0.1σ^3^. We find that the absence of effective threading results in a much faster transition to free diffusion and a 14-fold enhancement of diffusion coefficient (Figure 4D). This finding provides independent and unambiguous evidence that it is indeed the threadings between chains that are responsible for their correlated (subdiffusive) dynamics over short-intermediate times and resulting retarded center-of-mass diffusion. We note that, in dilute conditions, the dynamics of tadpoles does not depend on their design; this further confirms that the observed behavior is due to collective interactions (see SI Figure S11).

Finally, we mention that our results are in fair quantitative agreement with experiments^43^ (see SI) and that the zero-shear viscosity obtained from both, experimental and simulated tadpoles, is best fitted by a power law with exponent close to η_0_ ∼ *L*^4.5^. Nonetheless, the data also suggest that both experiments and simulations are performed in a crossover regime, and our analysis strongly supports the argument that in the asymptotic regime the tadpoles’ mobility should slow down exponentially in tail length (Figures 2B and 4).

In conclusion, we have investigated the dynamics of entangled systems of tadpole-shaped polymers, as the simplest example of a broader family of “chimeric” polymers formed by the combination of unknotted and unlinked loops and branches (Figure 1A). While similar architectures had been investigated in the dilute regime,^47,48^ here we design entangled systems with the aim of understanding how to achieve a fine control over threading topological constraints and, in turn, over the rheology of the bulk.

Here we have discovered that it is possible to design polymer architectures that can span a much larger dynamical range than that achievable with simpler architectures at fixed polymer mass. For instance, using tadpole-shaped polymers, we can explore a dynamical range that is about 2 orders of magnitude broader than the one for linear chains with modest lengths *N*/*N*_e_ = 25 (Figure 2D). Importantly, this phenomenon cannot be reproduced using ring–linear blends as their slowing down due to threading was observed to be of order unity compared with that of linear chains only^4,11,26,38^ and expected to scale only linearly with rings mass.^39^

We argue that this marked difference is due to the lack of a strategy to slow down linear chains more than reptation in ring–linear blends. On the contrary, the fused architecture of tadpoles (and of higher order chimeric polymers) together with the emergence of a hierarchical, percolating set of threading topological constraints entails that the process of constraint release imposed by linear tails on circular heads propagates back to tails too, causing a dramatic and system-wide slowing down. We feel it would be very interesting to directly compare the dynamics of tadpoles and that of ring–linear blends with same values of *C* and *L* in simulations and experiments.

By using minimal surfaces (Figure 3) and semiphantom interactions (Figure 4D), we unambiguously demonstrated that intertadpole threadings play a major role in the dynamics and that this effect is not due to single threadings (Figure 3B) but to correlated (Figure 3C) and collective (Figure 4C) ones. Interestingly, the more the threadings per tadpole, the slower is their full relaxation (Figure 4C), thus entailing further nonlinear slowing down in the large *N* limit (Figure 4B).

We have also showed that the relaxation of threadings can be mapped to that of a polydisperse system of polymers, with the caveat that here the distribution of threading lengths is uniform (Figure 3D) and that the exponent of the longest relaxation time increases with *L* (Figure 4A). This finding is in stark contrast with simpler architectures, e.g., linear, for which the relaxation exponent is insensitive on polymer length, e.g., δ = 3 for reptation of polymers with any *L*.

We argue that the phenomenology observed here might be generically expected across the broader family of chimeric polymers and that further fine-tuning can likely be achieved by varying the number of looped structures as well as their relative lengths. Ultimately, we envisage using these chimeric architectures to tune the dynamics of specific polymers that are expensive to synthesize in large scales. Our results suggest that even a modest polymer mass can display a broad dynamical range and this property can be harnessed to keep the costs low while achieving the desired rheology through informed polymer design. Our work might therefore serve to motivate future theoretical and experimental characterizations of entangled solutions of higher-order chimeric structures which may be now feasibly realized via synthetic chemistry^23,40,44^ or DNA origami.

## Supporting Information Available

Preparation of initial conformations of polymer melts, molecular dynamics computer simulations, numerical methods to analyze the simulations (PDF)
