# Supplementary material for: Threading-Induced Dynamical Transition in Tadpole-Shaped Polymers
Source: ACS Macro Lett. 2020 May 5;9(5):743–8. doi: 10.1021/acsmacrolett.0c00197 (PMC8016395; doi:10.1021/acsmacrolett.0c00197)
Supplement: Supplementary file 1 — mz0c00197_si_001.pdf [file mz0c00197_si_001.pdf]

# – Supporting Information –
Threading-Induced Dynamical Transition in Tadpole-Shaped Polymers

Angelo Rosa,[1,2] Jan Smrek,[3,2] Matthew S. Turner,[4,5] and Davide Michieletto[6,7,8]

[1] *Sissa (Scuola Internazionale Superiore di Studi Avanzati), Via Bonomea 265, 34136 Trieste, Italy*
E-mail: anrosa@sissa.it
[2] *Joint first authors*
[3] *Faculty of Physics, University of Vienna, Boltzmanngasse 5, A-1090 Vienna, Austria*
E-mail: jan.smrek@univie.ac.at
[4] *Department of Physics and Centre for Complexity Science,*
University of Warwick, Coventry, CV4 7AL, UK
[5] *Department of Chemical Engineering, Kyoto University, Kyoto, Japan*
E-mail: m.s.turner_kyoto@icloud.com
[6] *School of Physics and Astronomy, University of Edinburgh,*
Peter Guthrie Tait Road, Edinburgh, EH9 3FD, UK
[7] *MRC Human Genetics Unit, Institute of Genetics and Molecular Medicine,*
University of Edinburgh, Edinburgh EH4 2XU, UK
[8] *Department of Mathematical Sciences, University of Bath, North Rd, Bath BA2 7AY, UK*
E-mail: davide.michieletto@ed.ac.uk

MODELS AND METHODS

The polymer model

To model all polymer systems considered in this work, we resort to the computational framework considered in previous works on entangled polymer solutions [1–5]. It consists of a variant of the well known Kremer and Grest polymer model [6]. Excluded volume interactions between beads (including consecutive ones along the contour of the chains) are described by the shifted and truncated Lennard-Jones (LJ) potential:

$$U_{\rm LJ}(r) = \begin{cases} 4\epsilon \left[\left(\frac{\sigma}{r}\right)^{12} - \left(\frac{\sigma}{r}\right)^{6} + \frac{1}{4} \right] & r \le r_c \\ 0 & r > r_c \end{cases} , \quad (1)$$

where r denotes the separation between the bead centers. The cutoff distance $r_c = 2^{1/6}\sigma$ is chosen so that only the repulsive part of the Lennard-Jones is used. The energy scale is set by $\epsilon = \kappa_B T$ and the length scale by σ, both of which are set to unity in our simulations. Consistent with that, in this work all quantities are reported in reduced LJ units.

Nearest-neighbour monomers along the contour of the chains are connected by the finitely extensible nonlinear elastic (FENE) potential, given by:

$$U_{\rm FENE}(r) = \begin{cases} -0.5kR_0^2 \ln\left(1 - (r/R_0)^2\right) & r \le R_0 \\ \infty & r > R_0 \end{cases} , \quad (2)$$

where $k = 30\epsilon/\sigma^2$ is the spring constant and $R_0 = 1.5\sigma$ is the maximum extension of the elastic FENE bond.

In order to maximize mutual chain interpenetration at relatively moderate chain length [7] and hence reduce the computational effort, we introduce an additional bending energy penalty between consecutive triplets of neighbouring beads along the chains in order to control polymer stiffness:

$$U_{\rm bend}(\theta) = k_\theta \left(1 - \cos\theta\right) . \quad (3)$$

Here, θ is the angle formed between adjacent bonds, i.e. $\boldsymbol{t}_i \cdot \boldsymbol{t}_{i+1}/|\boldsymbol{t}_i||\boldsymbol{t}_{i+1}|$ with $\boldsymbol{t}_i$ the tangent at i, and $k_\theta = 5\kappa_B T$ is the bending constant. With this choice, the polymer is equivalent to a worm-like chain [8] with Kuhn length $\ell_K = 10\sigma$ [9].

Simulation details

We take polymer solutions consisting of M polymer chains, each polymer chain is made of N monomers and we consider chains with linear, circular or "tadpole" architecture, see Table SI for details. The total volume of the simulation box is fixed to $V = \frac{M\times N}{\rho}$ where the monomer number density $\rho\sigma^3 = 0.1$ as in previous works [1–5]. Since tadpoles $C = 250, L = 250$ in dilute conditions have gyration radius $R_g^2(dilute) \simeq 620\sigma^2$, we find that $c^* = 3N/4\pi R_g^3 \simeq 0.01\sigma^{-3}$, and hence we are working at about 10 times the overlap concentration.

The static and kinetic properties of the systems are studied using fixed-volume and constant-temperature Molecular Dynamics (MD) simulations with implicit solvent and periodic boundary conditions. MD simulations are performed using the LAMMPS package [11]. The equations of motion are integrated using a velocity Verlet algorithm, in which all beads are weakly coupled to a Langevin heat bath with a local damping constant $\Gamma = 0.5\tau_{\rm MD}^{-1}$ where $\tau_{\rm MD} = \sigma(m/\epsilon)^{1/2}$ is the Lennard-Jones time and $m = 1$ is the conventional mass unit. The integration time step is set to $\Delta t = 0.012\tau_{\rm MD}$.

Systems preparation, equilibration and productions runs

Preparation of the initial configurations

Linear polymers – Setting the stage for linear chains in solution is straightforward. Linear chains conformations were initially taken in the form of ordinary random walks and located at random positions within the simulation box. In order to remove unphysical overlaps between chain monomers, a short (of the order of a few $\tau_{\rm MD}$'s) Molecular Dynamics run with capped, soft (*i.e.* non-diverging) repulsive interactions between monomers was employed.

Chain topology	$N = C + L$	M	Equilibration time $[\times 10^7 \tau_{\rm MD}]$	$\langle R_g^2 \rangle [\sigma^2]$	$D[10^{-4}\sigma^2/\tau_{\rm MD}]$	$\eta_0[k_B T/\sigma^3 \tau_{\rm MD}]$
Linear ($C = 0$, $L = 67$)	67	600	0.12	78.48 ± 0.05	97.1 ± 0.3	$2.13 \pm 0.02^{(*)}$
Linear ($C = 0$, $L = 500$)	500	80	1.2	717.2 ± 15.4	1.4 ± 10^{-2}	428.1 ± 28.6
Ring ($C = 500$, $L = 0$)	500	80	1.2	237.6 ± 1.1	6.2 ± 10^{-2}	13.5 ± 0.7
Tadpole ($C = 100$, $L = 100$)	200	80	2.5	177.4 ± 0.7	10.7 ± 0.1	13.6 ± 0.4
Tadpole ($C = 100$, $L = 250$)	350	80	2.5	431.5 ± 6.2	1.06 ± 0.05	236.5 ± 29.0
Tadpole ($C = 100$, $L = 400$)	500	80	2.5	669.8 ± 24.9	0.278 ± 0.005	1477.4 ± 290.6
Tadpole ($C = 250$, $L = 100$)	350	120	2.4	222.3 ± 0.94	5.0 ± 0.005	22.4 ± 0.4
Tadpole ($C = 250$, $L = 250$)	500	80	2.5	459.1 ± 11.7	0.3 ± 0.005	516.0 ± 95.8
Tadpole ($C = 250$, $L = 400$)	650	70	6.0	763.6 ± 39.4	0.76 ± 0.005	3797.3 ± 987.6
Tadpole ($C = 400$, $L = 100$)	500	80	2.5	272.4 ± 1.7	2.7 ± 0.05	37.6 ± 2.5
Tadpole ($C = 400$, $L = 250$)	650	70	6.0	476.6 ± 12.2	0.15 ± 0.005	554.9 ± 25.7
Tadpole ($C = 400$, $L = 400$)	800	56	6.0	791.4 ± 36.7	0.04 ± 0.001	3998.9 ± 804.0

Table S I. Summary of the polymer systems considered in this work. Chain topology: total number of monomers in the head (C) and the tail (L) of the chain. $N = C + L$: total mass of the chain in number of monomers. M: total number of chains of the system. Equilibration time: length of the run to let the system to equilibrate after initial preparation of the sample (see section "Equilibration" for details). $\langle R_g^2 \rangle$: single chain mean-square gyration radius and corresponding error bars. D: single chain diffusion coefficient (defined assuming the asymptotic relationship $g_3(\tau) \equiv 6D\tau$ for large τ's) and corresponding error bars. η_0: zero-shear viscosity and corresponding error bars (see section "Comparison with experiments" for details). $^{(*)}$The value for linear chains ($C = 0, L = 67$) is used to normalize the numerical values of η_0 of the other systems, the number of entanglement lengths per chain ($L/L_e \approx 1.67$) having been chosen to match the shortest chains studied in the experimental work by Doi *et al.* [10].

Ring and "tadpole" polymers – For polymers displaying closed topology, i.e. ring or tadpole-shaped, we initialise the configurations in a very large box, *i.e.* at very dilute conditions, to prevent linking between the rings or the heads of the tadpoles. Next, we perform a short warm-up to gently displace overlapping atoms as done for linear chains. Following this short run, we turn on the FENE and LJ interactions which together prevent chain crossing [6] and we attain the correct monomer density of $\rho\sigma^3 = 0.1$ by performing a slow compression of the simulation box. At the end of this procedure, the pair-wise linking between rings and heads of tadpoles is checked once more.

Equilibration

Each system was let to equilibrate on a very long MD simulation of several tens of millions of $\tau_{\rm MD}$ (see Table SI for the exact values) during which each chain moves a spatial distance several times its own gyration radius R_g, see Fig. S1 illustrating a typical example.

Production runs

After equilibration, we have performed long runs of $2.5 \times 10^9 \Delta t = 3 \times 10^7 \tau_{\rm MD}$ for all systems, excepting $(C, L) = (250, 400), (400, 250)$ and $(400, 400)$ for which longer runs up to $12.5 \times 10^9 \Delta t = 1.5 \times 10^8 \tau_{\rm MD}$ were carried on. These runs have been used then for measuring the properties of the systems reported in this work. Single-chain properties (mean-square gyration radius, $\langle R_g^2 \rangle$, and diffusion coefficient, D) for the considered polymer solutions are summarized in Table SI.

Linear and Ring Polymers

To directly compare tadpoles with linear and ring polymers we have run new simulations of these architectures with $N = 500$ beads long (see Table SI). In addition, we report data from published ring [3] and unpublished linear polymers with contour lengths $256, 512$ and 1024 performed with slightly different values of friction coefficient and integration time-step. In particular we have used $\Gamma = \tau_{MD}^{-1}$ and $\Delta t = 0.01$. Since we do not expect these parameters to

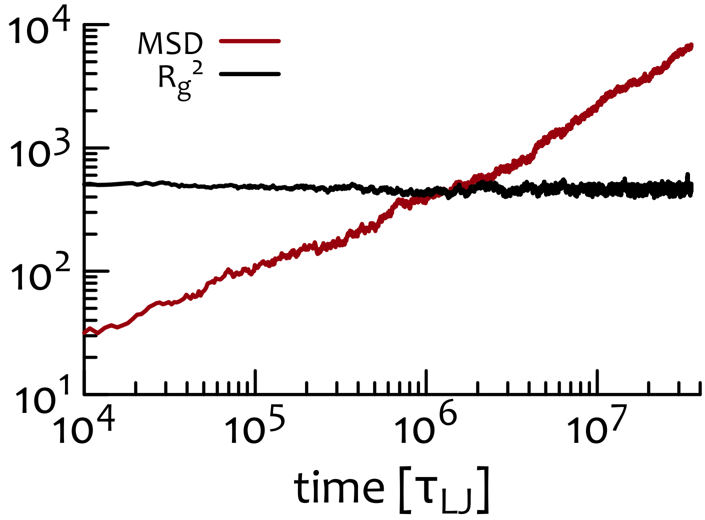

Fig. S 1. Equilibration run for tadpoles ($C = 250, L = 250$) showing that their centre of mass travels, on average, several times their size, R_g.

affect the scaling of statistical and dynamical quantities, we rescale the actual values of diffusion coefficient measured for $N = 512$ in order to match the one simulated here for $N = 500$. These data are shown in Fig. 2 of the main text and are employed to show the different scaling assumed by systems of tadpoles.

MEAN-SQUARE DISPLACEMENT AND DIFFUSION COEFFICIENT

We compute the mean-square displacement (MSD) of the tadpoles as

$$g_3(t) = \left\langle \frac{1}{T-t} \int_0^{T-t} \left[\boldsymbol{r}(t+t_0) - \boldsymbol{r}(t_0) \right]^2 dt_0 \right\rangle \tag{4}$$

with T the total observation time $T \gg t$ and $\boldsymbol{r}(t)$ the position of the centre of mass of the single chain at time t. The angular brackets indicate average over the system of M chains. In the main text we show the curves in units of σ^2 while here (Fig. S2A) we show them scaled by the mean-square chain size $\langle R_g^2 \rangle$. In Figs. S2B we report the values of the diffusion coefficient plotted against head length (in the main text we show against tail and total lengths).

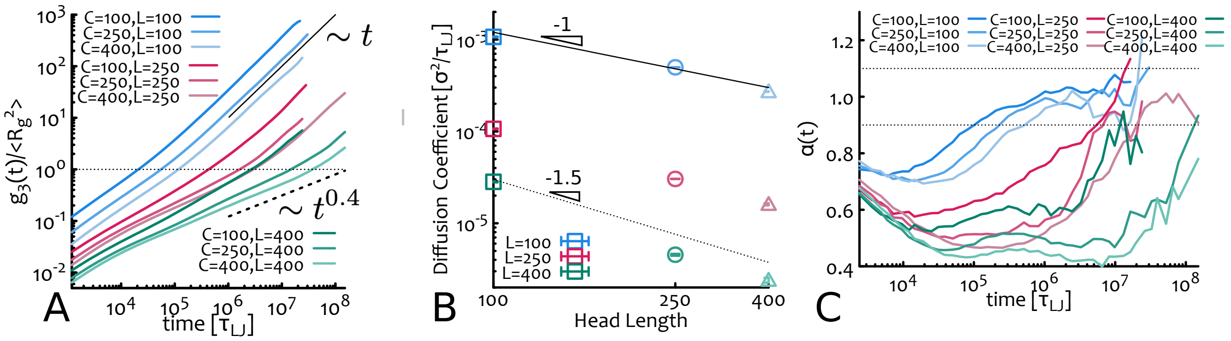

Fig. S 2. **Dynamics. A** Single-chain time MSD rescaled by tadpole mean-square size $\langle R_g^2 \rangle$. **B** Diffusion coefficient as a function of tail length for fixed head. Solid and dashed lines are guide for the eye. **C** Dynamical exponent α as a function of lagtime t. Dotted lines identify the region $\alpha = 1 \pm 0.1$ within with the fit of the MSD is performed.

The diffusion coefficient shown in Fig. S2B and main text is computed by fitting the large-time range of the MSD's with the function $f(t) = 6Dt^{\alpha}$, where α is constrained to be $= 1$ and D is the only free parameter. We validate if the

dynamical exponent $\alpha(t)$ becomes close to unity at large times by computing

$$\alpha(t) = \frac{d \log g_3}{d \log t}\,, \tag{5}$$

which is reported in Fig. S2C. As one can notice, at large times almost all the datasets fall within the range $\alpha = 1 \pm 0.1$ apart from $C = 400$, $L = 400$ for which we then interpret D as an upper bound value.

MINIMAL SURFACES

The construction of the minimal surface allows us to compute the minimal area A, which is expected to scale as $A \sim C$ in the limit of large contour length C [12]. In Fig. S3A we show that this scaling is well recapitulated by the tadpoles simulated here.

OTHER STATISTICS OF THREADINGS

A priori, each threading can be made of a number of "piercings"; each piercing defined as a single intersection of a tail with another tadpole's head. We can compute the statistics of piercings from the construction of minimal surfaces and in Fig. S3B we show that the distribution is exponential with a mean that is about 1 for all systems, meaning that irrespectively of tail length and head size, each threading is most of the times made up by a single piercing. As we show in the main text, this single intersection is roughly uniformly distributed along the tail and therefore the distribution of threading length is roughly constant. Some deviations from this trend is observed near the ends of the tail (see Fig 3D in the main text). In Fig. S3 we also report the average number of threadings per tadpole head i, i.e. $\phi_i(t) = \sum_{j=1(\neq i)}^{N} T_{ij}(t)$ averaged over tadpoles and over time $\langle \phi \rangle = 1/T \sum_{t=1}^{T} \left(1/N \sum_{i=1}^{N} \phi_i(t) \right)$.

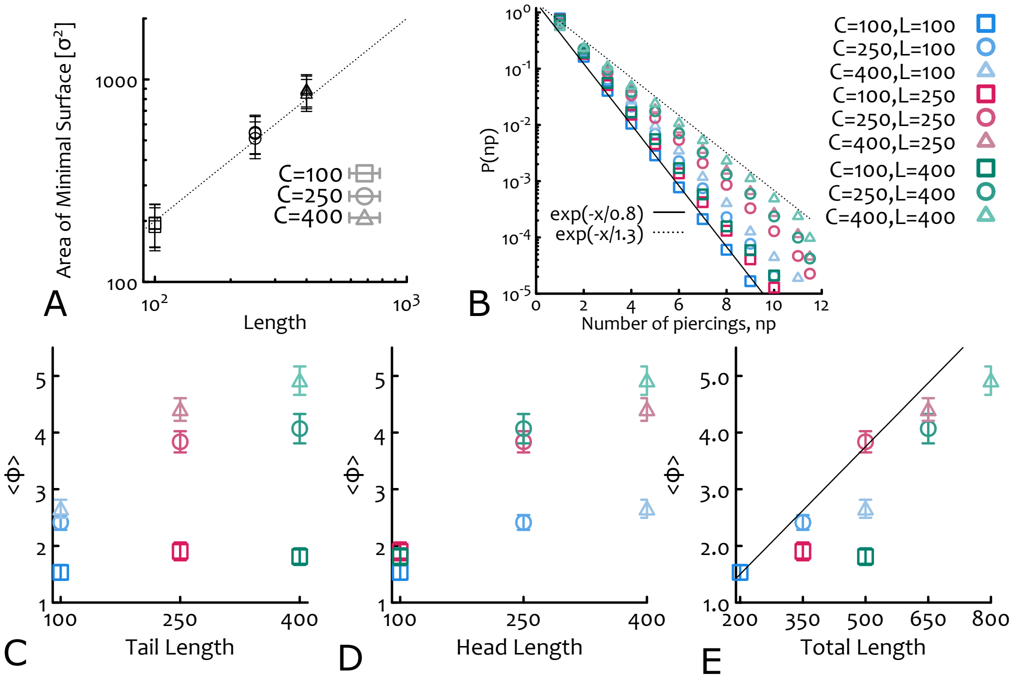

Fig. S 3. **Threading Statistics. A** The area of the minimal surface spanning the head of a tadpole scales roughly linearly with its head length C. Dashed line is a guide for the eye that scales linearly with length. **B** The distribution of piercings per threading is exponential with mean of about 1 piercing per threading for all systems. Solid and dashed lines are guides for the eye with mean 0.8 an 1.3 respectively. **C-E** Total number of threadings per head as a function of (**C**) tail length, (**D**) head length and (**E**) total contour length. The solid line in **E** is a guide for the eye and it scales linearly with the total contour length.

MSD OF THE TAIL-HEAD INTERSECTION

Through the minimal surface construction we can also quantify the dynamics of the bond intersecting the head-spanning surface, i.e. the dynamics of the each piercing. In practice, this is done by associating the piercing with the index of the closest bead along the tail. This index can be updated in time and it yields a mean-square displacement along the tail. As shown in Fig. S4, this dynamics is a subdiffusive walk, with an exponent compatible with Rouse dynamics, i.e. $\alpha \simeq 0.4 - 0.6$.

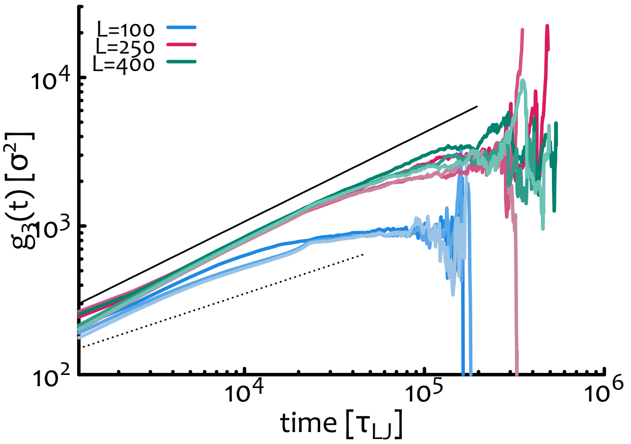

Fig. S 4. **MSD of piercings.** The MSD of each tail-head intersection displays a subdiffusive behaviour with $\alpha \simeq 0.4 - 0.6$ (dashed and solid lines respectively). Notice that the curves for $L = 100$ display an apparently slow dynamics because a piercing disappears on time scale comparable to the shortest lagtime probed ($1200\tau_{LJ}$) and a new one can appear nearby.

HEAD-HEAD THREADING PLAYS A MINOR CONTRIBUTION

The distribution of first unthreading times are broadly unaffected by inter-head threadings. We directly compare the two ways of calculating $\Theta(t)$, with and without head-head threading, in Fig. S5.

THREADING RELAXATION AS POLYDISPERSE PROBLEM

The relaxation of threadings can be recast in the problem of finding the global relaxation time in a system of polydisperse chains. This is due to the fact that the threadings involve fractions of the tails which do not have the same length, and we find that they are distributed uniformly (see main text) rather than exponentially as expected in a system of classic polydisperse polymers [13]. Hence one must solve a polydisperse relaxation problem with uniformly distributed polymer lengths. Considering only the largest relaxation mode we may compute

$$\tilde{\chi}(t) = \frac{1}{L}\int_0^L \exp\left[-\left(\frac{t}{\tau_0 l^\delta}\right)\right] dl\,, \tag{6}$$

where the threading relaxation depends on the length of the threading l as $T(l) = \tau_0 l^\delta$. We numerically integrate Eq. (6) for fixed τ_0, δ and L, find a theoretical function $\tilde{\chi}(t)$, fit it to the curves $\chi(t)$ computed from the simulations and find the best fitting values of τ_0 and δ for fixed L and C. The behaviour of δ is reported in the main text and grows continuously with L. We stress that for a system of classic linear polymers one would find that δ is constant and equal to 2 for Rouse dynamics and 3 for reptation [13]. At the same time, we expect a roughly constant value of τ_0, being this a microscopic relaxation time. This is reported in Fig. S6 and it is indeed roughly constant in L.

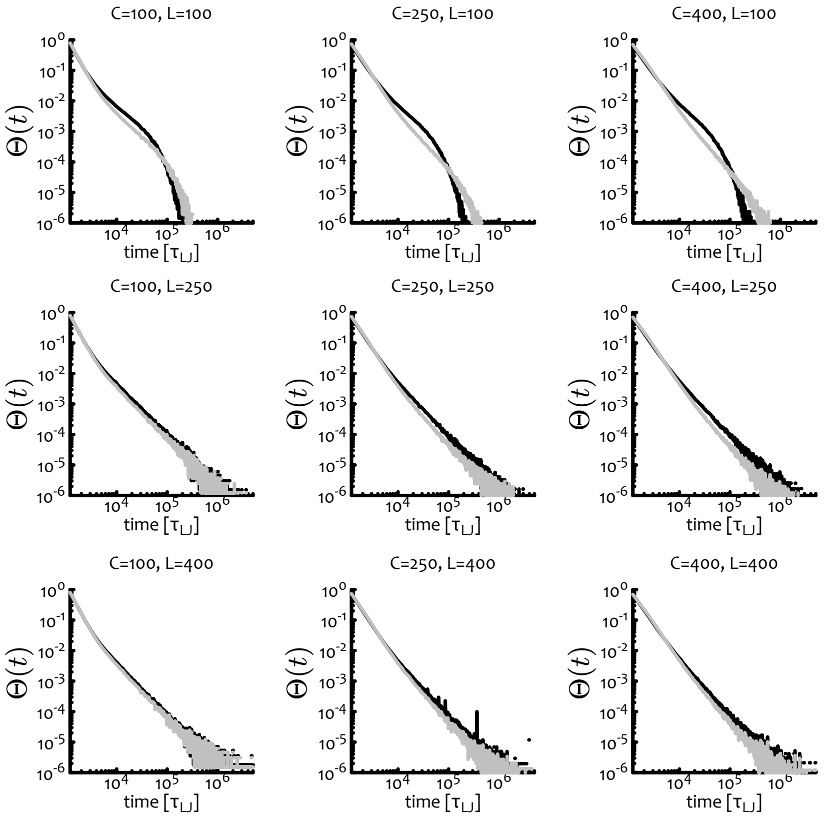

Fig. S 5. **Comparison of first unthreading distribution.** Direct comparison of the distribution of first unthreading time $\Theta(t)$ as reported in the main text (Fig. 3) with (grey) and without (black) accounting for head-head threadings for all systems. The designs (values of C and L) are reported at the top of each panel. As one can notice the deviation is small for most of the systems.

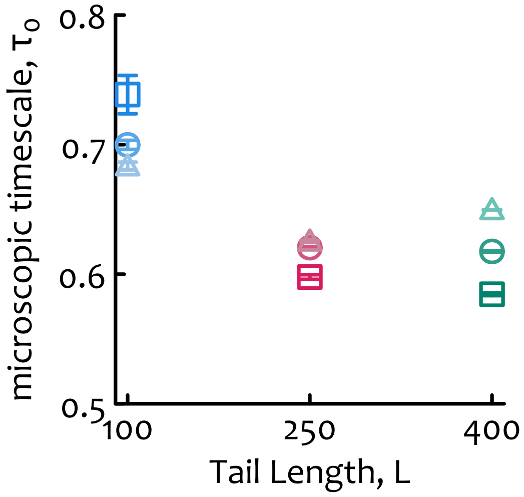

Fig. S 6. **Microscopic Threading Relaxation Timescale.** This plot shows that the microscopic timescale τ_0 in the function $T(l) = \tau_0 l^{\delta}$ is only weakly dependent on L, as expected.

CORRELATION BETWEEN NUMBER OF THREADINGS AND DYNAMICS

In this section we report plots to show how the dynamics of the system, e.g. diffusion coefficient D, depends on threading statistics. We find that it is difficult to unambiguously understand the role played by threadings on the behaviour of D versus $\langle\phi\rangle$, because both depend (differently) on the total contour length and on the size of ring and linear architectures (see Fig. S7). This figure further testifies that the impact of threadings on the dynamics is highly non-trivial.

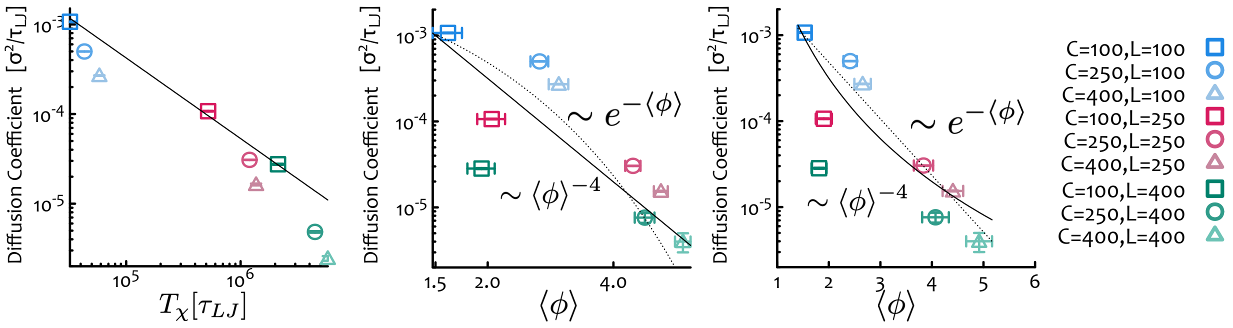

Fig. S 7. **Correlation between diffusion coefficient and threadings.** (left) Diffusion coefficient versus T_χ roughly following a power law decay T_χ^{-1}. The solid line is a guide for the eye. (centre) Diffusion coefficient versus average number of threadings per tadpole in log-log plot. (right) Same as central plot, this time in a semi-log plot. An exponential and power law (with exponent -4) decays are shown as guides for the eye.

PERCOLATING NETWORK

In Fig. 4B of the main text we show that the average number of threadings per tadpole $\langle\phi\rangle$ is always larger than unity, also for the shortest chains. This strongly suggests that the threadings create a network among tadpoles that is percolating. To confirm this conjecture, we have now analysed the structure of the threading network in the following way. We consider two tadpoles to belong to the same threading cluster if at least one of them threads the other one. When we look at all possible threadings, we observe that all tadpoles belong to one cluster, for all the studied systems i.e. the network is percolating and the largest connected component $M_{\max}$ nearly equals the total number of tadpoles M (Fig. S8A).

However, our method allows us also to discriminate the threadings based on their length, i.e. the contour length of tail from its tip to the last intersection (closest to the head) with the minimal surface (or the only one in the case of only one piercing). Therefore, we can also decide to consider two rings to belong to the same cluster if they are threading with a threading length at most some chosen l_{cutoff}. We can thus compute the number of tadpoles in the largest connected component of the threaded network ($M_{\max}/M$) while varying l_{cutoff} (Fig. S8A).

As can be seen from the figure, if l_{cutoff} is small enough only a few threadings are taken into account and the threading network is not percolating, i.e. the number of tadpoles belonging to the largest connected component $M_{\max}$ is not comparable with the total number of tadpoles M. For larger l_{cutoff} (and indeed when we consider threadings of any length ($l_{\text{cutoff}} = L$) the network is percolating, i.e. $M_{\max} \sim M$. [Note that here we are showing that percolation is occurring on an abstract graph where tadpoles are nodes rather than spatial percolation across the physical simulation box.]

An interesting observation is that the transition (say at $M_{\max}/M = 0.5$) is at small $l_{\text{cutoff}} \simeq 0.1L$ for the larger tadpoles, while at $\simeq 0.5L$ for the tadpoles with short tail ($L = 100$) and small head ($C = 100$). This has a nice explanation in terms of our previous results: since the distribution of threading lengths is roughly uniform (Fig. 3D), the $\langle\phi\rangle$ threadings (on average) through any one tadpole (Fig. 4B) will uniformly populate the lengths $[0 : L]$. At the same time, by choosing a cutoff length we are effectively considering only a fraction of the total threadings per tadpole, i.e. $N_t(l) = l\langle\phi\rangle/L$ (this equality holds because they are uniformly distributed in lengths). Now, we recall that for a network to be percolating one requires that the average number of connections (threadings in this case) is larger than some critical value $N_t(l) \geq n_c$. Hence, one arrives at the conclusion that the cutoff on the threading length that must be imposed to "destroy" the percolating network is $N_t(l_{\text{cutoff}}) = n_c$ or $l_{\text{cutoff}}/L = n_c/\langle\phi\rangle$.

Another way to define percolation is by fixing an arbitrary threshold on $M_{\max}/M = m$ and by finding the value of

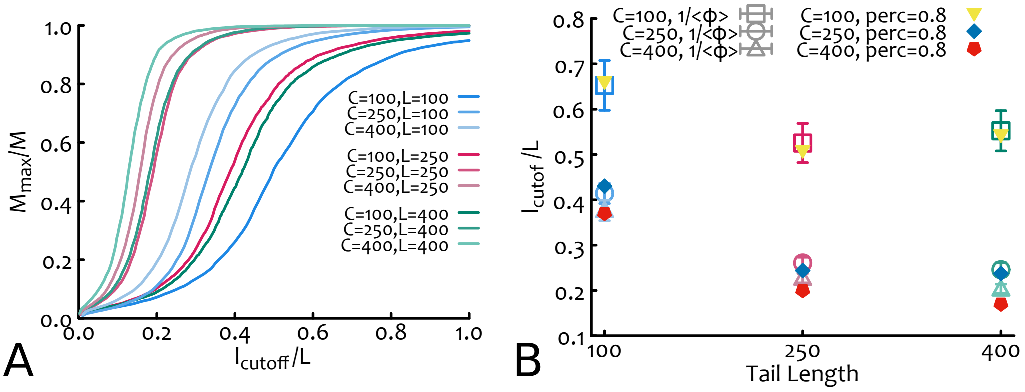

Fig. S 8. **A.** Size of the largest connected component normalised by the total number of tadpoles, for different values of the threading length $l_{\rm cutoff}/L$. **B.** Comparing the values of $1/\langle\phi\rangle$ and the critical value of $l_{\rm cutoff}/L$ at which $M_{\rm max}/M = 0.8$.

$l_{\rm cutoff}/L$ that meets this threshold. Since we don't know the details of the underlying network, we cannot unambiguously fix values for n_c or m for which the network is unambiguously percolating. Nonetheless, we find that for any choice of $n_c \lesssim 1$ there exist a corresponding value of m for which $l_{\rm cutoff}/L$ is matching the former and that is robust across all systems. For instance, in Fig. 8B we show the case $n_c = 1$, $m = 0.8$ and plot $l_{\rm cutoff}/L = 1/\langle\phi\rangle$ in the same plot together with the root of $M_{\rm max}/M(l_{\rm cutoff}/L) = 0.8$. One can notice that the agreement is excellent. Different choices of m and n_c yield similar plots.

Finally, we highlight that Fig. 8 shows two different "branches" (or groups of curves): one for small heads and one for large ones and that nicely mirror their dynamics as described in the main paper.

COMPARISON WITH EXPERIMENTS

In this section we discuss the comparison with the experiments performed by Doi and colleagues on tadpole-shaped polystyrene polymers [10]. In their work they consider one fixed head size (about 3.3 entanglement lengths) and variable tail length 1.6, 3.9 and 6.6 entanglement lengths. They measure the loss and storage moduli $G'(w)$ and $G''(w)$ and from these, the zero-shear viscosity

$$\eta_0 = \lim_{w\to 0} \frac{G''(w)}{w} \, . \tag{7}$$

They find a very slow relaxation of the tadpoles that is comparable with that of stars polymers, i.e. η_0 increases exponentially with tail length. To compare their results with ours, we compute the stress relaxation modulus $G(t)$ as

$$G^{\alpha\beta}(t) = V\langle\sigma^{\alpha\beta}(t)\sigma^{\alpha\beta}(0)\rangle/k_BT \tag{8}$$

where

$$\sigma^{\alpha\beta}(t) = \sum_{k,l} F^{\alpha}_{kl}(t) r^{\beta}_{kl}(t) \tag{9}$$

is the pre-averaged stress tensor [14, 15]. Latin indexes run over monomers in the system, Greek ones over components, r is the distance between beads k and l and F the force between these beads. $G(t)$ curves are reported in Fig. S9. Then, from $G(t)$ we compute the zero-shear viscosity $\eta_0 \equiv \int_0^\infty G(t)dt$ [8] by estimating numerically the integral employing a finite upper bound

$$\eta_0 \approx \int_0^{\tau_d} G(t)dt \, , \tag{10}$$

which is defined as the time for the chain to diffuse over a distance equal to its own size, $g_3(\tau_d) \equiv \langle R_g^2\rangle$. Error bars were estimated by taking the mean-square deviation of $xy-$, $xz-$ and $yz-$ contributions to η_0 (Eq. (8)) from the corresponding average. The values of η_0 for the different systems considered in this work are summarized in Table SI and represented in Fig. S10 together with the experimental values from Ref. [10].

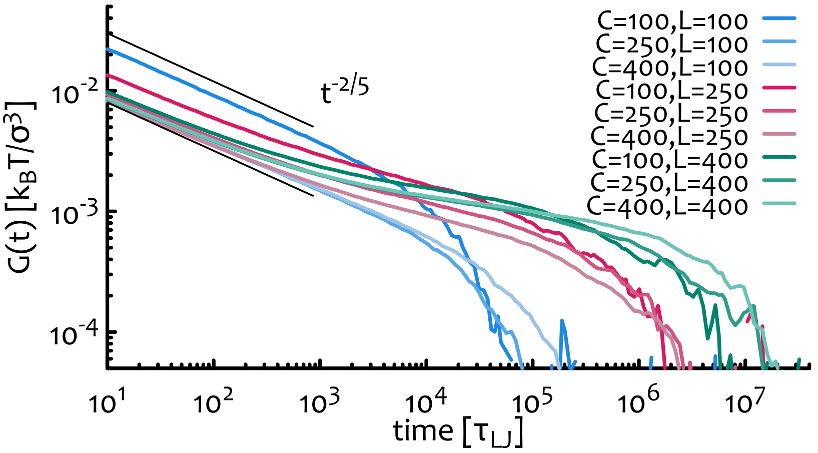

Fig. S 9. **Stress Relaxation Curves.** Stress relaxation computed as per Eq. (8) for different tadpole designs. The scaling at early times is compatible with that of ring polymers [16], i.e. $G(t) \sim t^{-2/5}$.

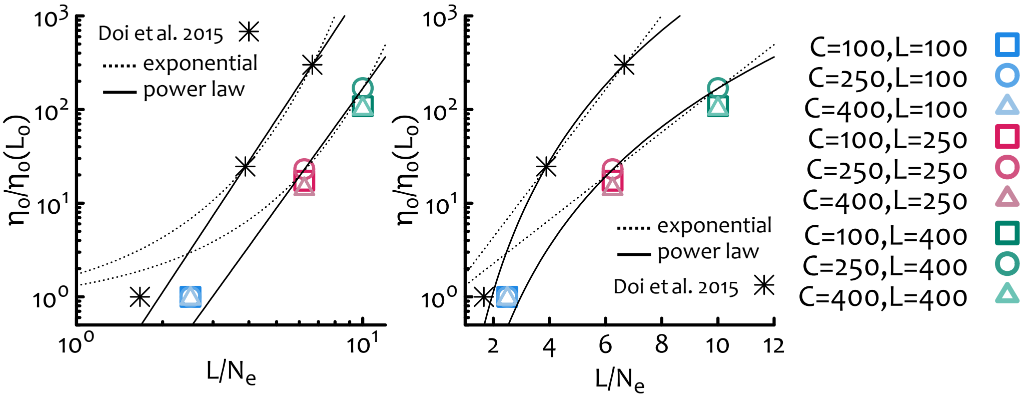

Fig. S 10. **Comparison with Experimental Data – Dependence on Tail Length.** The zero shear viscosity is plotted against tail length (normalised by entanglement length in experiments (18 kg/mol [10] and simulations $N_e = 40\sigma$ [1]) in log-log (left) and semilog (right) plot. To compare with experiments in the same plot, we rescale the value of η_0 for that measured in the case of shortest tails ($\eta_0(L_0)$). This rescaling is completely arbitrary, nevertheless it does not affect the functional behaviour of the data. Fits corresponding to exponentials and power laws are shown as dashed and solid lines respectively. The χ^2 test is in both cases (experiments vs simulations) smaller using power law functions $f(x) = ax^b$. The best fit is obtained with exponents $b = 4.64(1)$ for experiments and $b = 4.24(1)$ for simulations.

To make a meaningful comparison, we show the lengths normalised by the entanglement length in the respective systems and the zero shear viscosity normalised by that attained by the system with smallest value of tail length (denoted as L_0 in the plots). We note that both sets of data are compatible with power law and exponential behaviour as a function of tail length and it appears that the range of values considered by us and by Doi and colleagues sit within a crossover region. We perform a fit of both data sets with power law and exponential function and we find that the χ^2 test returns smaller values when considering power laws in both cases. The exponent that we find for the simulated systems is very close to the one fitting experimental data, i.e. about 4.5 (see Fig. S10).

Instead of arbitrarily normalising the value of η_0 by that attained by the system with shortest tails (for a fixed head), we can also normalise it by the value of η_0 displayed by a system of linear polymers 1.67 entanglement lengths, as this value is provided in Ref. [10]. By performing this normalisation we find that our simulations are in fair quantitative agreement with experiments on linear chains (see top panel of Fig. S11). We then use the same value of viscosities to normalise the tadpoles data and find that our dataset is again in fair agreement with the only data point available for symmetric tadpoles from Ref. [10] (see bottom panel of Fig. S11).

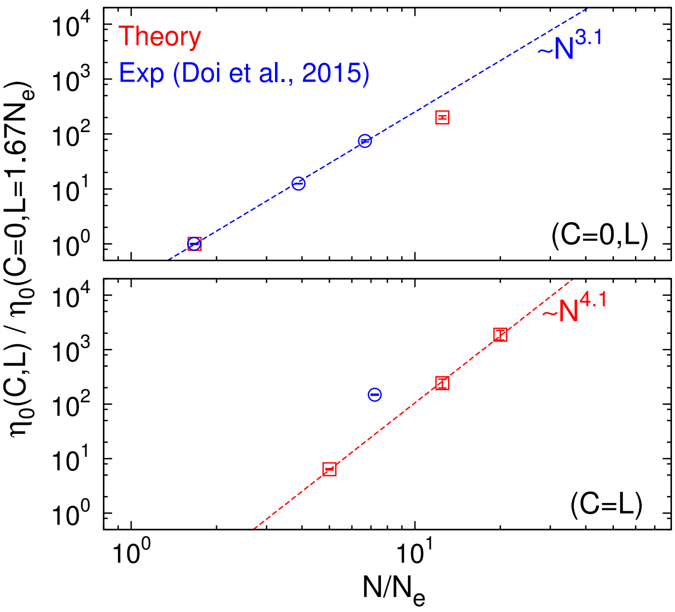

Fig. S 11. **Comparison with Experimental Data – Dependence on Total Length.** The zero shear viscosity is plotted against the total polymer contour length, N, normalised by the entanglement length N_e ($N_e = 40\sigma$ in simulations and $N_e = 18$ kg/mol in experiments [10]). The value of η_0 is here normalised by that measured for systems of linear chains $C = 0$ with length $L = 1.67N_e$ in both experiments and simulations. Results from our simulations are shown in red squares while the values from experiments are shown in blue circles. (Top) Case $C = 0$, i.e. linear polymers; (bottom) $C = L$, i.e. symmetric tadpoles. Notice the larger exponent found in simulations of tadpoles which would be interesting to test in experiments.

DYNAMICS OF TADPOLES IN DILUTE CONDITIONS

In this section we report simulations of tadpoles with different designs performed in dilute conditions. Specifically we consider $N = 5$ tadpoles, $M = 500$ beads each at monomer density $\rho = NM/L^3 = 0.001\sigma^{-3}$, 100 times smaller than considered up to now. We perform 10 independent simulations of this system and calculate the time and ensemble averaged MSD of the centre of mass, which is reported in Fig. S12. As one can notice there is no dependence of the MSD on the tadpole design, proving that the effects observed in dense solutions (main text) are genuinely arising from collective interactions. This result was to be expected as our simulations do not take into account hydrodynamic interactions as these are strongly suppressed in entangled solutions, the regime on which we focus on in this work. We note that in Ref. [17] the authors account for hydrodynamic interactions in dilute conditions and report different dynamics depending on the design of “topological polymers”, e.g. tadpoles, theta, etc.

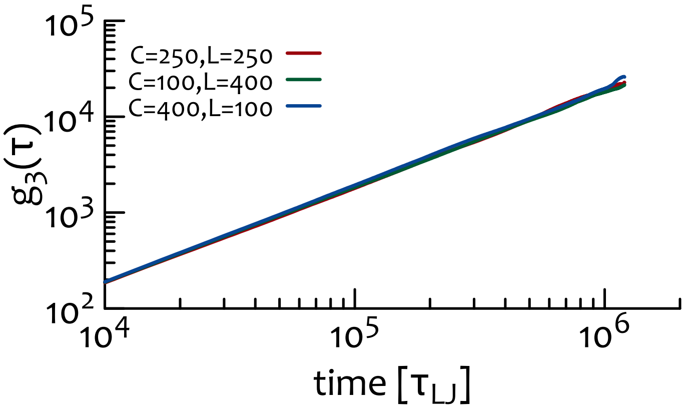

Fig. S 12. **The slowing down is due to collective interactions.** MSD of tadpoles with fixed total length $N = 500$ and in dilute conditions do not display a dependence on the design.

[1] A. Rosa and R. Everaers, "Ring Polymers in the Melt State: The Physics of Crumpling," Phys. Rev. Lett. **112**, 118302 (2014).
[2] N. Nahali and A. Rosa, "Density Effects in Entangled Solutions of Linear and Ring Polymers," J. Phys.: Condens. Matter **28**, 065101 (2016).
[3] D. Michieletto and M. S. Turner, "A Topologically Driven Glass in Ring Polymers," Proc. Natl. Acad. Sci. USA **113**, 5195–5200 (2016).
[4] D. Michieletto, N. Nahali, and A. Rosa, "Glassiness and Heterogeneous Dynamics in Dense Solutions of Ring Polymers," Phys. Rev. Lett. **119**, 1–18 (2017).
[5] N. Nahali and A. Rosa, "Nanoprobe Diffusion in Entangled Polymer Solutions: Linear vs. Unconcatenated Ring Chains," J. Chem. Phys. **148**, 194902 (2018).
[6] K. Kremer and G. S. Grest, "Dynamics of Entangled Linear Polymer Melts: A Molecular-Dynamics Simulation," J. Chem. Phys. **92**, 5057 (1990).
[7] M. Müller, J. P. Wittmer, and M. E. Cates, "Topological Effects in Ring Polymers. II. Influence of Persistence Length," Phys. Rev. E **61**, 4078–89 (2000).
[8] M. Rubinstein and R. H. Colby, *Polymer Physics* (Oxford University Press, New York, 2003).
[9] R. Auhl, R. Everaers, G. S. Grest, K. Kremer, and S. J. Plimpton, "Equilibration of Long Chain Polymer Melts in Computer Simulations," J. Chem. Phys. **119**, 12718–12728 (2003).
[10] Y. Doi, A. Takano, Y. Takahashi, and Y. Matsushita, "Melt Rheology of Tadpole-Shaped Polystyrenes," Macromolecules **48**, 8667–8674 (2015).
[11] S. Plimpton, "Fast Parallel Algorithms for Short-Range Molecular Dynamics," J. Comp. Phys. **117**, 1–19 (1995).
[12] J. Smrek and A. Y. Grosberg, "Minimal Surfaces on Unconcatenated Polymer Rings in Melt," ACS Macro Lett. **5**, 750–754 (2016).
[13] P.-G. De Gennes, "Relaxation Anomalies in Linear Polymer Melts," Macromolecules **35**, 3785–3786 (2002).
[14] W. B. Lee and K. Kremer, "Entangled Polymer Melts: Relation between Plateau Modulus and Stress Autocorrelation Function," Macromolecules **42**, 6270–6276 (2009).
[15] J. Ramírez, S. K. Sukumaran, B. Vorselaars, and A. E. Likhtman, "Efficient on the Fly Calculation of Time Correlation Functions in Computer Simulations," J . Chem. Phys. **133**, 154103 (2010).
[16] M. Kapnistos, M. Lang, D. Vlassopoulos, W. Pyckhout-Hintzen, D. Richter, D. Cho, T. Chang, and M. Rubinstein, "Unexpected Power-Law Stress Relaxation of Entangled Ring Polymers," Nat. Mater. **7**, 997–1002 (2008).
[17] E. Uehara and T. Deguchi, "Statistical and Hydrodynamic Properties of Topological Polymers for Various Graphs Showing Enhanced Short-Range Correlation," J. Chem. Phys. **145**, 164905 (2016).
